# Optimality Driven Nearest Centroid Classification from Genomic Data

**DOI:** 10.1371/journal.pone.0001002

**Published:** 2007-10-03

**Authors:** Alan R. Dabney, John D. Storey

**Affiliations:** 1 Department of Statistics, Texas A&M University, College Station, Texas, United States of America; 2 Department of Biostatistics, University of Washington, Seattle, Washington, United States of America; 3 Department of Genome Sciences, University of Washington, Seattle, Washington, United States of America; University of Michigan, United States of America

## Abstract

Nearest-centroid classifiers have recently been successfully employed in high-dimensional applications, such as in genomics. A necessary step when building a classifier for high-dimensional data is feature selection. Feature selection is frequently carried out by computing univariate scores for each feature individually, without consideration for how a subset of features performs as a whole. We introduce a new feature selection approach for high-dimensional nearest centroid classifiers that instead is based on the theoretically optimal choice of a given number of features, which we determine directly here. This allows us to develop a new greedy algorithm to estimate this optimal nearest-centroid classifier with a given number of features. In addition, whereas the centroids are usually formed from maximum likelihood estimates, we investigate the applicability of high-dimensional shrinkage estimates of centroids. We apply the proposed method to clinical classification based on gene-expression microarrays, demonstrating that the proposed method can outperform existing nearest centroid classifiers.

## Introduction

Linear Discriminant Analysis (LDA) is a long-standing prediction method that has been well characterized when the number of features used for prediction is small [1]. The method has recently been shown to compare favorably with more complicated classifiers in high-dimensional applications, where there are thousands of potential features to employ, but only a subset are used [2], [3]. In the LDA setting, each class is characterized by its vector of average feature values (i.e., class centroid). A new observation is evaluated by computing the scaled distance between its profile and each class centroid. The observation is then assigned to the class to which it is nearest, allowing LDA to be interpreted as a “nearest centroid classifier.”

In high-dimensional applications, it is often desirable to build a classifier using only a subset of features due to the fact that (i) many of the features are not informative for classification and (ii) the number of training samples available for building the classifier is substantially smaller than the number of possible features. It can also be argued that a classifier built with a smaller number of features is preferable to an equally accurate classifier built with the complete set of features. This problem is analagous to, but in general distinct from, that of selecting variables in a regression model by, say, least angle regression (LARS) [4]. Early work on the feature selection problem in discriminant analysis has been summarized elsewhere [5], [6].

Several approaches have been recently proposed for nearest centroid classifiers that rely on univariate statistics for feature selection [2], [7]–[9]. These methods assess each feature individually by its ability to discriminate the classes. However, it has been noted that the features that best discriminate the classes individually are not necessarily the ones that work best *together* [10]. In the extremely simple case where features are uncorrelated and only two classes exist, it intuitively follows that the optimal set of features are those whose means are most different between the two classes. However, this intuition does not easily carry over to the more complicated case where features are correlated and/or there are more than two classes. For example, if we seek to classify among three classes, it has not been shown whether it is better to choose features that distinguish one class from the other two well or those that distinguish among all three classes well. The role of correlation between features is not currently well understood either.

In this paper, we provide a theoretical result showing how to determine the subset of features of a given size that minimizes the misclassification rate for a nearest-centroid classifier. For example, if 800 features are available, but one wants to build a classifier consisting of 12 features, we show which 12 provide the lowest misclassification rate. This optimal feature set takes into account the joint behavior of the features in two ways. First, it explicitly incorporates information about correlation between features. Second, it assesses how a group of features as a whole is capable of distinguishing between multiple classes. While we show how to define the theoretically optimal subset, we must *estimate* the optimal subset in practice. The benefit of characterizing the theoretically optimal target, and the novelty of our contribution, is that we provide the optimal criteria for comparing subsets. This reflects the basic motivation for the work presented here, to identify the theoretically optimal solution to the feature selection problem, then try to get as close to this solution as possible. For practical implementation, we propose a simple greedy algorithm for searching subsets and demonstrate its operating characteristics.

Two existing papers [2], [11] propose univariate feature selection techniques to build maximum likelihood estimate based nearest-centroid classifiers, the latter of which is called the “Classification to Nearest Centroids” (Clanc) method. The method proposed here is related to these, except that we propose a multivariate feature selection method and consider parameter estimation using a multivariate shrinkage technique.

Nearest centroid classifiers have been shown to perform well with gene-expression microarrays [2], [3], and we illustrate our findings in this setting. We compare our proposed classifier with existing nearest centroid classifiers in extensive simulations and on three previously-published microarray datasets. Our results demonstrate that improvements in prediction accuracy can be attained by estimating the optimal feature-selection criteria.

## Methods

### Background

An LDA classifier is a canonical nearest centroid classifier. The problem it addresses is to classify unknown samples into one of *K* classes. To build a classifier, we obtain *n_k_* training samples per class, with *m* features per sample. For each training sample, we observe class membership *Y* and profile *X*. For simplicity, we will represent the classes by the numbers *k*  =  1,2,…,*K*. Note that each profile is a vector of length *m*. We assume that profiles from class *k* are distributed as *N*(μ*_k_*,Σ), the multivariate normal distribution with mean vector μ*_k_* and covariance matrix Σ. Call *L*(*x*;μ*_k_*,Σ), the corresponding probability density function. Finally, let π*_k_* be the prior probability that an unknown sample comes from class *k*.

Bayes' Theorem states that the probability that an observed sample comes from class *k* is proportional to the product of the class density and prior probability:
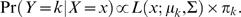
(1)We call Pr(Y = *k*|*X* = *x*) the posterior probability that sample *x* comes from class *k*. LDA assigns the sample to the class with the largest posterior probability. This can be shown to be the rule that minimizes misclassification error [1]. The rule can be written as:
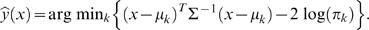
(2)Thus, a sample is assigned to the class to which it is nearest, as measured by the metric 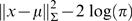, where 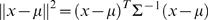 is the square of the Mahalanobis distance between *x* and μ.

### Optimal Nearest Centroid Classifiers

#### Misclassification rates

A misclassification occurs when a sample is assigned to the incorrect class. The probability of making a classification error is:
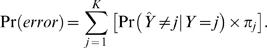
(3)We can derive misclassification rates using the LDA rule in equation (2). In particular, we can calculate misclassification rates for any subset of features. An optimal subset can be found by simply assigning misclassification rates to all possible subsets of a given size and choosing the one with the lowest error rate.

The misclassification rate of a nearest-centroid (LDA) classifier can be shown to be
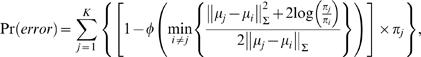
(4)where φ is the *cdf* of the standard normal distribution, and 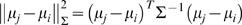 is the square of the Mahalanobis distance between μ*_j_* and μ*_i_*; note that this assumes the data are Normally distributed, as stated by the model. The subset of features of size *m*_0_≤*m* that minimizes the misclassification rate is the one with the lowest value of equation (4); note that the *m*−*m*_0_ features not included in the subset are not involved in the calculation. This defines the optimal subset of size *m*_0_.

Equation (4) can be interpreted as measuring the collective distance between all of the class centroids. In general, the misclassification rate will be small when all of the class centroids are far away from each other. Note, however, that the score in (4) is actually a complicated combination of the pairwise differences between the centroids and the class priors. Furthermore, correlations between features are explicitly incorporated through the distance functions ||μ*_j_*−μ*_i_*||_Σ_. Further intuition into (4) can be attained by considering the following simple example.

#### A Simple Example

The data in Table 1 represent an artificial example with 10 features and 3 classes. The population means of each class are shown in columns two through four; we assume that each feature has variance 1 and that all features are uncorrelated. Suppose that we wish to select the five features that correspond to the lowest misclassification rate. The final column of the table lists univariate scores for each feature, where we have used the average squared difference from the overall feature mean as the score. A high value for a feature on this score indicates large overall differences between this feature's class means. The five largest univariate scores correspond to features 1, 2, 3, 4, and 5.

**Table 1:** Class means with 10 features and 3 classes.

Feature	μ_1_	μ_2_	μ_2_	Score
1	3.00	0.00	0.00	2.00
2	2.00	0.00	0.00	0.89
3	1.50	0.00	0.00	0.50
4	1.25	0.00	0.00	0.35
5	0.00	1.10	0.00	0.27
6	0.00	1.00	0.00	0.22
7	0.00	0.90	0.00	0.18
8	0.00	0.00	0.85	0.16
9	0.00	0.00	0.75	0.12
10	0.00	0.00	0.65	0.09

An alternative approach to using univariate scores to select features is to consider all 252 possible quintuplets and choose the set with the lowest overall misclassification rate. Note that, to do this, we must be able to assign misclassification probabilities to arbitrary feature subsets. This highlights the utility of the multivariate score (4). Using (4), we find that the set of features chosen by the univariate scores has an overall misclassification rate of 20%. Similarly, we find that the optimal set in this example contains features 1, 5, 6, 7, and 8, with an associated error rate of 13%. The most obvious difference between this subset and that chosen by univariate scores is the exclusion of features 2, 3, and 4. Apparently, class one can be sufficiently characterized by feature 1. The other features do not contain sufficient *additional* information to merit their selection. We note in this example that the optimal subset of size *m*_0_ + 1 contains the optimal subset of size *m*_0_, *m*_0_ = 1, 2,…,9, although this need not be true in general.

#### Correlation Between Features

An important aspect of the optimal feature-selection procedure is its explicit incorporation of correlation between features. It is not necessarily clear what effect correlation between features should have on a classifier. Intuitively, many weakly informative, correlated genes might be expected to collectively be highly informative. However, it has been shown [12] that estimating correlations as zero, making Σ a diagonal matrix, can lead to better prediction when the number of features is large relative to the number of samples.

We investigate the effect of different correlation patterns in the example of Table 1. Let Σ be the 10×10 covariance matrix. In Table 2, we refer to the 4×4 block corresponding to features 1–4 as “Block 1.” The blocks corresponding to features 5–7 and 8–10 are similarly referred to as “Block 2” and “Block 3,” respectively. “Block 1∼2” refers to the blocks specific to both features 1–4 and features 5–7, *etc.* In all cases, the selected block is given an autoregressive covariance structure with correlation parameter 0.9, meaning that the correlation between two correlated features *i* and *j* is 0.9^|*i*-*j*|^; no qualitative differences were found when considering negative correlation. We report the best subset under each correlation pattern (*S_O_*), its error rate, and the error rate for the best subset chosen ignoring correlation (*S_I_*). We also report the rank of the best subset ignoring correlation in each case.

**Table 2:** The effect of covariance on the optimal feature-selection procedure.

		Error		
Covariance	Selected Features	*S_O_*	*S_I_*	Rank *S_I_*
None	1, 5, 6, 7, 8	13.1%	13.1%	1
Block 1	1, 5, 6, 7, 8	13.1%	13.1%	1
Block 2	1, 5, 8, 9, 10	14.9%	18.2%	64
Block 3	1, 5, 6, 7, 8	13.1%	13.1%	1
Block 1∼2	1, 4, 5, 8, 9	6.1%	14.0%	143
Block 1∼3	1, 5, 6, 7, 8	11.6%	11.6%	1
Block 2∼3	1, 2, 3, 7, 8	2.2%	3.5%	18

In this example, correlations that affect features 5–7 change the entries of the best subset, as well as the associated error rates. For example, when there is correlation within features 5–7, the optimal subset includes features 1, 5, 8, 9, and 10, with an error rate of 14.9%. The set chosen ignoring correlation ranks 64th (out of 252 possible subsets), with an error rate of 18.2%. These results suggest that correlated features can be useful together. While there are many possible scenarios in which correlation could play a role, the main point is that the feature-selection procedure guided by (4) automatically identifies the optimal combination of features, even in the presence of correlation. Of course, in practice, there is the added challenge of estimating the class centroids and covariance matrix. In particular, when there are many more features than samples, it is not clear that covariances can be estimated well enough to make them worth the effort. We consider this further in the next section.

### Proposed Nearest Centroid Classifier

In practice, unknown model parameters and the general impracticality of exhaustive searches with genomic data preclude our finding theoretically optimal subsets. Instead, we must *estimate* optimal subsets. A myriad of solutions for this problem have been proposed, particularly in the context of gene expression microarrays. The novelty of our proposed method is that we have provided the ideal target for estimation. Specifically, our goal in practice is to choose the subset that minimizes an estimated version of (4). To avoid the need for exhaustive searches, we propose a greedy search algorithm.

We consider several variations of our basic algorithm, employing different methods for estimating class centroids and covariance matrices. We compare the proposals with existing alternatives on both simulated and real datasets. Details of the proposed algorithms are given in what follows. Based on our comparisons, the final algorithm that we propose for nearest-centroid classification from genomic data uses shrunken centroids and a diagonal covariance matrix. The following is the proposed algorithm to build a classifier with *m*_0_ features:

1. Estimate the class centroids 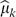 by simple averaging using equation (5).
2. Estimate the pooled variances using equation (8) to form the diagonal covariance estimate 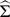.
3. Using the estimated centroids and variances from Steps 1 and 2, find the single feature with the smallest estimated misclassification rate from equation (4).
4. For *b* = 2,3,…,*m*_0_, consider each remaining feature separately:
  - Combine one remaining feature with the already selected feature(s) 1,2,…,*b*−1, and use these to form shrunken class centroid estimates 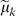 based on equations (6–7).
  - Find the single feature with the smallest estimated misclassification rate according to equation (4), when included with the already-selected feature(s) 1,2,…,*b*−1.
5. The final nearest centroid classifier is composed of the subsets of 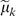 and 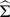 built from the *m*_0_ selected features.

We call this method “Clanc”, as it is an extension of the Clanc procedure proposed earlier [11]. The algorithm has also been implemented in the Clanc software [13].

#### Estimating the Decision Rule

To estimate (4), we estimate the class centroids μ*_k_*, *k* = 1,2,…,*K*, and the common covariance matrix Σ. Reducing the MSE of each estimated centroid will bring us closer to (4) [11]. According to Stein's Paradox of statistics [14], we can reduce the MSEs by shrinking each centroid toward its overall mean (or any other constant). In our setting, this suggests shrinking each centroid estimate across its *m* components. We note that existing shrinkage proposals for nearest-centroid classifiers shrink each feature across the *K* classes [9]. This makes the estimated centroids less distinguishable and tends to result in *increased* misclassification rates [11].

While there are many possible approaches to shrinking the centroids, we take the following simple approach. We begin with the usual centroid estimate 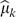, an *m*-vector with *i*th component equal to the average expression for feature *i* in class *k*:
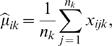
(5)where *x_ijk_* is the expression for feature *i* in sample *j* of class *k*. Now let 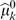 be the *m*-vector with each of the *i* = 1,2,…,*m* components equal to the estimated overall mean for class *k*, 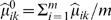, and consider shrunken centroids of the form
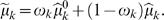
(6)We choose ω*_k_* so that 
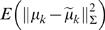
 is minimized. It can be shown that the desired value of ω*_k_* is
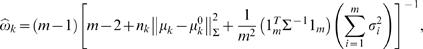
(7)where 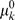 is the *m*-vector with each component equal to the overall mean for class *k*, 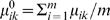, 1*_m_* is the *m*-vector of ones, and the 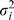 are the diagonal components of Σ. In practice, we use plug-in estimates for the unknown parameters.

Drawing from earlier versions of our work [15], a recent article has also considered the use of shrinkage across genes rather than across centroids [16]. Their shrinkage proposal is based on hard-thresholding. By contrast, we propose the shrinkage of each centroid toward a constant, as was originally considered by Stein [14]. Furthermore, the eigenvector-based selection routine proposed in reference [16] does not estimate the optimal selection, as we do here.

While we can form an unbiased estimate of the covariance matrix Σ, such an estimate will tend to be singular in the microarray setting, with many more features than samples. Furthermore, theoretical justification for shrinking the off-diagonal components of Σ to zero in such settings has recently been published [12]. An alternative approach is to attempt to improve our estimate of Σ by shrinkage. Shrunken covariance matrices can be of the form 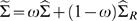. Here, 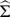 is the usual unbiased empirical covariance matrix estimate, 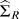 is the estimate under some simplifying restriction (such as diagonal form), and ω is a constant that is used to multiply every matrix entry. For example, 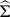 has (*i*, *j*)th element
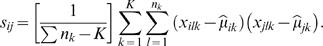
(8)


This is a pooled version of the class-specific estimates, reflecting the model assumption of a common covariance matrix. In what follows, we define 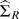 as the diagonalized version of 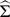, with diagonal elements equal to the diagonal elements of 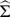 and all off-diagonal elements set to zero.

The shrinkage parameter ω can be estimated using cross-validation on training data [17] or by computing the value that minimizes mean square error (MSE) [18]. We follow the latter approach in the results below, as described in Table 1 of reference [18]. Specifically, denote the (*i*, *j*)th off-diagonal element of the covariance matrix as 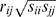, where *s_ii_* and *r_ij_* are the empirical variance and correlation, respectively. Shrinkage is applied to the correlation parameters, with the shrunken version of *r_ij_* equal to
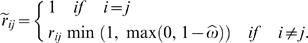
(9)The estimator 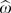 proposed in reference [18] that approximately minimizes the MSE of the shrunken covariance matrix is
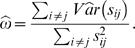
(10)Adapting the estimator in reference [18] to our pooled covariance matrix setting, we have
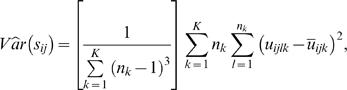
(11)where 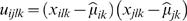 and 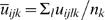.

#### Searching for the Optimal Subset

We have characterized the theoretically optimal subset of given size by computing the misclassification rate (4) for nearest-centroid classifiers. Ideally, having estimated the decision rule, we would evaluate all subsets of given size and choose the one corresponding to the lowest estimated error rate. In high-dimensional settings, such an exhaustive search is not feasible. We now consider practical strategies for searching for the optimal subset. These are analogous to existing routines for selecting subsets in discriminant analysis [5], [6].

A simple approach to this problem is to rank each feature individually on its ability to discriminate between the classes and choose the top features from this list. Many previous publications use versions of univariate *t*-statistics or *F*-statistics to score features [2], [7]–[9], [11]. As discussed above, univariate selection procedures do not take into account the joint performance of a set of features. Thus, whereas the selected features will each *individually* discriminate well, the selected *set* of features may not.

As an alternative to univariate scoring, we might consider more computationally-intensive search algorithms. For example, a greedy forward-selection algorithm would (i) select the one feature that scores best individually according to some criterion, (ii) select the one feature that scores best together with the already chosen feature(s), (iii) repeat until the desired number of features have been chosen. The misclassification rate (4) itself is the ideal score to use for guiding the selection of a subset. As such, we propose the greedy forward-selection algorithm that proceeds as above, using an estimate of (4) to score each proposed subset. For reference, the greedy algorithm identifies the same subset as the exhaustive search algorithm in the example of Table 1.

## Results

### Illustration on Simulated Examples

We evaluate different methods for estimating the optimal subset of a given size with the following sets of simulations. There are 3 classes and 1000 genes, from which a subset of size 30 is desired. For each class, 15 training samples and 15 test samples are generated. In simulation set one (Table 3), the class centroids are equidistant from one another. In simulation set two (Table 4), class one is more easily distinguished than class two, which is more easily distinguished than class three. The second scenario is analogous to that described in Table 1. A univariate scoring procedure will prefer features from class one, since its features will score highest individually. A forward-selection procedure, on the other hand, will spread out the chosen features among the classes in such a way that the overall misclassification rate is approximately minimized. We also consider the effect of correlation among the features. Since the misclassification rate (4) explicitly incorporates correlations, we expect that using (4) to guide feature selection will achieve greater accuracy relative to its competitors.

**Table 3:** Test error rates (standard errors): Classes equidistant.

			Absolute Value of Correlation							
Algorithm	Centroids	Covariance	0.00		0.40		0.65		0.90	
PAM	shrunken*	diagonal	0.15	(0.07)	0.15	(0.06)	0.18	(0.07)	0.29	(0.08)
Clanc	unshrunken	unrestricted	0.30	(0.08)	0.28	(0.08)	0.28	(0.07)	0.11	(0.07)
Clanc	shrunken	unrestricted	0.27	(0.09)	0.27	(0.08)	0.26	(0.10)	0.09	(0.07)
Clanc	unshrunk.	diagonal	0.07	(0.04)	0.08	(0.04)	0.10	(0.04)	0.19	(0.07)
Clanc	shrunken	diagonal	0.06	(0.04)	0.08	(0.04)	0.10	(0.05)	0.19	(0.07)
Clanc	unshrunk.	shrunken	0.30	(0.08)	0.28	(0.08)	0.28	(0.07)	0.06	(0.04)
Clanc	shrunken	shrunken	0.27	(0.09)	0.27	(0.08)	0.26	(0.10)	0.06	(0.04)

* Shrinkage in this case takes place across classes rather than across features.

**Table 4:** Test error rates (standard errors): Classes not equidistant.

			Absolute Value of Correlation							
Algorithm	Centroids	Covariance	0.00		0.40		0.65		0.90	
PAM	shrunken*	diagonal	0.33	(0.02)	0.33	(0.02)	0.34	(0.03)	0.37	(0.03)
Clanc	unshrunken	unrestricted	0.25	(0.07)	0.26	(0.08)	0.25	(0.08)	0.12	(0.08)
Clanc	shrunken	unrestricted	0.25	(0.08)	0.28	(0.09)	0.22	(0.07)	0.13	(0.09)
Clanc	unshrunk.	diagonal	0.04	(0.03)	0.04	(0.03)	0.06	(0.04)	0.14	(0.06)
Clanc	shrunken	diagonal	0.03	(0.03)	0.03	(0.03)	0.06	(0.03)	0.13	(0.06)
Clanc	unshrunk.	shrunken	0.25	(0.07)	0.26	(0.08)	0.25	(0.08)	0.12	(0.08)
Clanc	shrunken	shrunken	0.25	(0.08)	0.28	(0.09)	0.22	(0.07)	0.13	(0.09)

* Shrinkage in this case takes place across classes rather than across features.

The details of the simulations are as follows. Twenty five percent of the genes are noise, with centroid components μ*_i_*_1_ = μ*_i_*_2_ = μ*_i_*_3_ = 0. Another 25% of the genes characterize class one, with centroid components μ*_i_*_1_ = 0.5 or 1 in simulation sets one and two, respectively, and μ*_i_*_2_ = μ*_i_*_3_ = 0. Another 25% of the genes characterize class two, with μ*_i_*_2_ = 0.5 and μ*_i_*_1_ = μ*_i_*_3_ = 0. The remaining genes characterize class three, with μ*_i_*_3_ = 0.5 or 0.25 in simulation sets one and two, respectively, and μ*_i_*_1_ = μ*_i_*_2_ = 0. In each simulation, the genes are randomly broken into 50 blocks of 20 genes. Within each block, an autoregressive covariance structure is used. Correlation (ρ) is positive in half of the blocks and negative in the other half. In each simulation set, four scenarios are presented: (i) independence (ρ = 0), (ii) low correlation (ρ = 0.4), (iii) medium correlation (ρ = 0.65), and (iv) high correlation (ρ = 0.9). Samples for class *k* are generated from *N_m_*(μ*_k_*,Σ), the multivariate normal distributions with mean vector μ*_k_* and covariance matrix Σ.

For each of 50 simulations, we applied the following nearest centroid classification methods. We report the PAM method [9], which uses univariate statistics to score features individually, and several variants of our greedy algorithm. We evaluate the effect of shrinking the centroids using equations (6–7). We also evaluate different choices for the covariance matrix: unrestricted, diagonal, and shrunken [18]. Precise expressions for each covariance matrix choice are given in equations (8–11).

Table 3 reports the results for simulation set one, and Table 4 is for simulation set two. The second column indicates whether the centroids were shrunken; PAM is marked with an asterisk in these Tables due to its alternative approach to shrinking centroids across classes rather than across features. The third column indicates the form of the covariance matrix used. The remaining numbers report test error rates, averaged across the 50 simulations, for each of the considered levels of correlation. The numbers in parentheses next to the error rates are the estimated standard errors. The Clanc classifiers that estimate nonzero covariances increase in accuracy with higher levels of correlation. While slightly less accurate with high correlations, Clanc using a diagonal covariance matrix performs well in all simulations. When classes are equidistant, the classifiers based on univariate scoring perform well. When classes are not equidistant, a substantial increase in accuracy can be had by employing a greedy search. There is some evidence that using shrunken centroids improves accuracy.

### Illustration on Real Examples

We now illustrate our methods on three previously published gene-expression microarray experiments. We compare the methods on the basis of error rates from five-fold cross-validation. We avoid gene-selection bias by completely rebuilding classifiers to identical specifications in each cross-validation iteration [19]. Cross-validated error rates are nearly unbiased, being slightly conservative, and they are thus sufficient for comparing classifiers. Note that the optimal subset depends on the prior probabilities π*_k_*, *k* = 1, 2,…, *K*. In what follows, we assume equal priors, although no substantial changes were seen when using priors that reflected the proportions observed in the samples.

The first example involves small round blue cell tumors (SRBCT) of childhood [20]. Expression measurements were made on 2,307 genes in 83 SRBCT samples. The tumors were classified as Burkitt lymphoma, Ewing sarcoma, neuroblastoma, or rhabdomyosarcoma. There are 11, 29, 18, and 25 samples in each respective class. In the second example, expression measurements were made on 4,026 genes in 58 lymphoma patients [21]. The tumors were classified as diffuse large B-cell lymphoma and leukemia, follicular lymphoma, and chronic lymphocytic leukemia. There are 42, 6, and 10 samples in each respective class. The third example involves the cell lines used in the National Cancer Institute's screen for anti-cancer drugs [22]. Expression measurements were made on 6,830 genes in 60 cell tumors. There are representative cell lines for each of lung cancer, prostate cancer, CNS, colon cancer, leukemia, melanoma, NSCLC, ovarian cancer, renal cancer, and one unknown sample. We filtered out 988 genes for which 20% or more of the tumors had missing values. We also excluded samples from prostate cancer (due to there being only two samples) and the one unknown sample. There are 9, 6, 7, 6, 8, 7, 6, and 8 samples in each remaining respective class.

**Figure 1:**
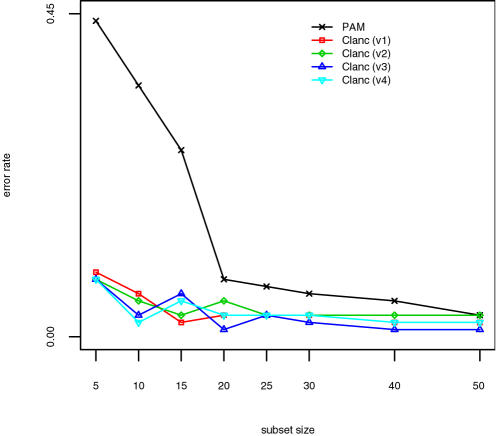
Results for SRBCT data. Classifiers are identical to those in Tables 3 and 4, with Clanc v1-v4 corresponding to the last four variants reported there, respectively.

**Figure 2:**
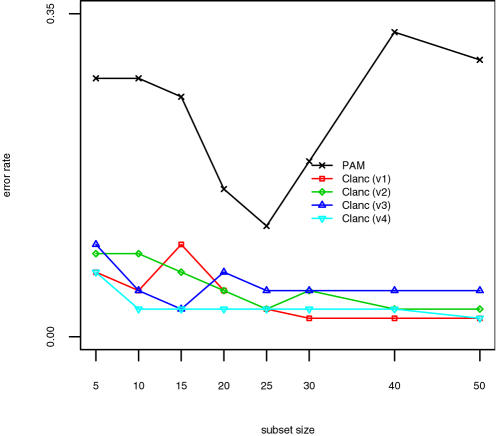
Results for Lymphoma data. Classifiers are identical to those in Tables 3 and 4, with Clanc v1-v4 corresponding to the last four variants reported there, respectively.

**Figure 3:**
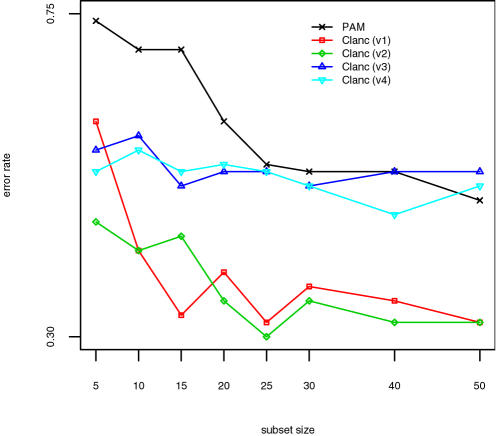
Results for NCI data. Classifiers are identical to those in Tables 3 and 4, with Clanc v1-v4 corresponding to the last four variants reported there, respectively.

The results for the SRBCT data are shown in Figure 1, those for the lymphoma data in Figure 2, and those for the NCI data in Figure 3. The classifiers presented are identical to those in Tables 3 and 4, except that Clanc classifiers with unrestricted covariances are excluded. The Clanc classifiers indicated by “v1-v4” correspond to the last four classifiers reported in Tables 3 and 4. Clanc improves accuracy over the PAM approach using univariate scoring. Shrunken centroids in Clanc improve accuracy in the NCI example but make no difference in the other examples. Diagonal covariance matrices result in greater accuracy overall for these examples. Overall, we interpret these results as indicating that Clanc classifiers with greedy searches guided by (4) can outperform the existing PAM classification method. In particular, the results support the use of shrunken centroids and diagonal covariance matrices, and we have implemented this algorithm in the Clanc software [13].

## Discussion

We have characterized the theoretically optimal subset of a given size for a nearest centroid classifier. We have also considered the estimation of this optimal subset. Although an exhaustive search would be ideal, it is not generally practical in the genomic setting. We have thus proposed a greedy algorithm for estimating optimal subsets and demonstrated that the resulting classifier can produce more accurate classifiers in both simulated and real applications. Our results indicate that some improvement in accuracy can be had by shrinking class centroids, for which we have proposed a novel procedure. Although the theoretically optimal subset explicitly incorporates correlation between features, our results concur with those of others in suggesting that correlations should be shrunken to zero in settings with many more features than samples.

We note that our approach to estimating the optimal decision rule could likely be improved upon. In particular, while MLE estimators of the class centroids and common covariance matrix themselves have good properties, the resulting estimator of the decision rule may not. An alternative in the two class case would be to directly estimate the decision rule using a variant of logistic regression. The multiclass case would be more complicated. We intend to investigate these issues in future work.
